# Bexarotene treatment does not clear β-Amyloid in an AD mouse model and Beagle dogs

**DOI:** 10.1186/1750-1326-8-S1-P40

**Published:** 2013-09-13

**Authors:** Ina Tesseur, Adrian Lo, Anouk Roberfroid, Sofie Dietvorst, Bianca Van Broeck, Marianne Borgers, Harrie Gijsen, Diederik Moechars, Marc Mercken, John Kemp, Rudi D’Hooge, Bart De Strooper

**Affiliations:** 1 VIB Center for the Biology of Disease, Leuven, Belgium; 2 KU Leuven and Universitaire Ziekenhuizen, Center for Human Genetics and Institute of Neuroscience & Disease (LIND), Leuven, Belgium; 3 Laboratory of Biological Psychology and Leuven Institute of Neuroscience & Disease (LIND), University of Leuven, Leuven, Belgium; 4 Neuroscience Department, Janssen Research and Development, a Division of Janssen Pharmaceutica NV, Beerse, Belgium

## Background

In our aging society Alzheimer’s Disease (AD) is becoming more and more prevalent while effective symptomatic therapeutics remain limited and no cure is available. ApoE4 is the most important genetic risk factor for AD. Previous results by Cramer *et al. *[1] showed that Bexarotene treatment reduced Aβ in the brain of wild type and AD model mice via an apoE-mediated clearance mechanism. Bexarotene, an RXR agonist, is an FDA approved drug for cutaneous T cell lymphoma and hence a preferred candidate for clinical testing. In this study we attempted to replicate the data by Cramer *et al. *[1].

## Methods

We used captisol^®^ (Cydex Pharmaceuticals), and HP-b-CD/ Tween to dissolve Bexarotene for administration in mice and dogs respectively. For acute experiments we administered a single 100 mg/kg/p.o. dose of Bexarotene (Ontario Chemical, Inc., Canada) to wild type male Swiss CD1 mice and measured endogenous levels of soluble Aβ_x-37_, Aβ_x-38_, Aβ_x-40_ and Aβ_x-42_ in brain at different time points. In Beagle dogs we administered 25 and 100 mg/kg/p.o. Bexarotene and measured Aβ_x-37_, Aβ_x-38_, Aβ_x-40_ and Aβ_x-42_ levels in CSF. For chronic experiments we administered 100 mg/kg/day/p.o. Bexarotene for 19 days to 10-months-old male hAPP/PS1 mice.

## Results

In contrast to the published data, we found that acute and chronic treatment with Bexarotene had no significant effects on Aβ levels in the brain of wild type and AD model mice and in Beagle dogs, despite high penetration of the drug into the brain. Although behavioral alterations were observed in AD model mice, adverse effects of the treatment confounded these observations.

## Conclusions

Drug formulation and pharmacokinetics might explain our contradictory observations with Cramer *et al. *[1] at least partly. These issues need to be resolved before Bexarotene can be tested in AD.

## References

[B1] Cramer PE Cirrito JR ApoE-directed therapeutics rapidly clear β-amyloid and reverse deficits in AD mouse models Science 2012 335 1503 1506 10.1126/science.1217697 22323736 PMC3651582

